# Supplementary material for: Pulmonary function following hyperbaric oxygen therapy: A longitudinal observational study
Source: PLoS One. 2023 May 31;18(5):e0285830. doi: 10.1371/journal.pone.0285830 (PMC10231819; doi:10.1371/journal.pone.0285830)
Supplement: S1 Table — Hyperbaric oxygen therapy indications approved by Health Canada (*) or the US Food and Drug Administration (†). Unlabeled items are approved by both agencies. (DOCX) [file pone.0285830.s001.docx]

**S1 Table**

| Air or Gas Embolism |
| --- |
| Carbon Monoxide Poisoning |
| Gas Gangrene |
| Compartment Syndrome, Crush Injury, or other Traumatic Ischemias |
| Decompression Sickness |
| Non-Healing Wounds |
| Severe Anemia |
| Intracranial Abscess* |
| Necrotizing Soft Tissue Infection |
| Osteomyelitis |
| Delayed Radiation Injury |
| Compromised Skin Grafts and Flaps |
| Thermal Burn Injury |
| Idiopathic Sudden Sensorineural Hearing Loss |
| Sudden Painless Vision Loss^†^ |
