# Supplementary material for: Pulmonary function following hyperbaric oxygen therapy: A longitudinal observational study
Source: PLoS One. 2023 May 31;18(5):e0285830. doi: 10.1371/journal.pone.0285830 (PMC10231819; doi:10.1371/journal.pone.0285830)
Supplement: S3 Table — Data resulting from a secondary analysis of the full cohort, which evaluated interval change in pulmonary function test performance between study timepoints. Abbreviations: FEV1% = percentage of predicted forced expiration volume in one second; FVC% = percentage of predicted forced vital capacity; FEF25-75% = percentage of predicted mid-expiratory flow; DE = difference estimate; LCL = lower confidence limit; UCL = upper confidence limit. (DOCX) [file pone.0285830.s003.docx]

**S3 Table**

| **Measure** | **Intervals** | **DE** | **LCL** | **UCL** |
| --- | --- | --- | --- | --- |
| FEV1% | (Pre-HBOT) - 20 | 2.07 | -1.24 | 5.38 |
|  | (Pre-HBOT) - 40 | 0.61 | -3.32 | 4.55 |
|  | (Pre-HBOT) - 60 | 3.39 | -4.02 | 10.80 |
|  | 20 - 40 | -1.45 | -5.47 | 2.56 |
|  | 20 - 60 | 1.32 | -6.14 | 8.78 |
|  | 40 - 60 | 2.77 | -4.89 | 10.4 |
| FVC% | (Pre-HBOT) - 20 | 1.91 | -0.92 | 4.75 |
|  | (Pre-HBOT) - 40 | -0.32 | -3.69 | 3.05 |
|  | (Pre-HBOT) - 60 | 2.97 | -3.38 | 9.33 |
|  | 20 - 40 | -2.24 | -5.68 | 1.21 |
|  | 20 - 60 | 1.06 | -5.33 | 7.46 |
|  | 40 - 60 | 3.30 | -3.28 | 9.87 |
| FEF25-75% | (Pre-HBOT) - 20 | 0.16 | -3.99 | 4.31 |
|  | (Pre-HBOT) - 40 | 0.64 | -4.30 | 5.58 |
|  | (Pre-HBOT) - 60 | 2.26 | -7.06 | 11.58 |
|  | 20 - 40 | 0.48 | -4.56 | 5.53 |
|  | 20 - 60 | 2.10 | -7.28 | 11.48 |
|  | 40 - 60 | 1.62 | -8.02 | 11.26 |
